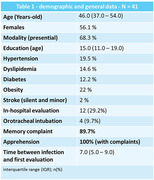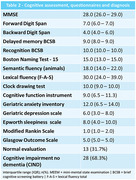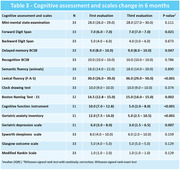# Cognitive profile of post‐COVID19 syndrome patients after cognitive rehabilitation on a six‐month follow‐up study in a public Brazilian center

**DOI:** 10.1002/alz.091999

**Published:** 2025-01-03

**Authors:** Raphael Ribeiro Spera, Raphael de Luca e Tuma, Júlia Chartouni Rodrigues, Satiko Andrezza Takano Peixoto, Adalberto Studart Neto, Eliane C Miotto, Artur Martins Coutinho, Sonia Maria Dozzi Brucki

**Affiliations:** ^1^ Medical School of University of São Paulo, São Paulo, São Paulo Brazil; ^2^ Hospital das Clínicas, Faculdade de Medicina da Universidade de São Paulo, São Paulo, São Paulo Brazil; ^3^ Behavioral and Cognitive Neurology Unit of the Neurology Department, University of São Paulo, São Paulo Brazil; ^4^ University of São Paulo Medical School, São Paulo Brazil; ^5^ University of São Paulo, São Paulo Brazil; ^6^ Hospital das Clínicas of University of São Paulo Medical School, Sao Paulo Brazil; ^7^ University of São Paulo School of Medicine, São Paulo, São Paulo Brazil

## Abstract

**Background:**

Post‐COVID19 syndrome is characterized by signs and symptoms that occur within 3 months of the onset of COVID19 acute phase and last at least 2 months. In the past 3 years, cognitive impairment has frequently been associated with COVID19 with descriptions of attentional, executive, memory, and language disorders. Many studies have assessed these cognitive disturbances using online and telephone tests, often in isolated interviews on a cross‐sectional design in high‐income countries. Few studies evaluated the role of cognitive rehabilitation in this situation.

**Method:**

Objective: To characterize the cognitive profile and psychological batteries of this population pre‐and post‐cognitive rehabilitation after 6 months. We selected patients younger than 65 years of age with COVID19 and cognitive symptoms for more than 3 and less than 6 months and no records of previous neurodegenerative or severe psychiatric diseases. They were evaluated 3 times (0, 3, and 6 months) with cognitive battery tests and questionnaires and later by a neuropsychologist for evaluation and cognitive training.

**Result:**

46 patients were recruited; 41 matched the inclusion criteria and were referred to cognitive rehabilitation. 33 finished the third medical assessment. General and demographic data are shown in **Table 1**. Cognitive assessment in the first evaluation is demonstrated in **Table 2**. Only 32% of the sample had an unremarkable evaluation, and 68% were classified as having Cognitive impairment no dementia (CIND). **Table 3** demonstrates patients that completed the follow‐up and the changes in cognitive tests at 6 months after rehabilitation, showing a statistically significant (p<0,05) improvement on some specific tests and questionnaires: Forward digit span, delayed memory on brief cognitive screening battery, lexical fluency (F‐A‐S), Boston naming test‐15, cognitive function instrument, geriatric anxiety inventory, and geriatric depression scale.

**Conclusion:**

Post‐COVID‐19 syndrome and cognitive impairment are relatively common also in mild infection and healthy adult patients. After cognitive rehabilitation, we depicted significant results in attention, episodic memory, verbal fluency, and psychiatric symptoms.